# Single-Cell Transcriptomic Analysis of Ecosystems in Papillary Thyroid Carcinoma Progression

**DOI:** 10.3389/fendo.2021.729565

**Published:** 2021-11-01

**Authors:** Ting Yan, Wangwang Qiu, Huaiyu Weng, Youben Fan, Guangwen Zhou, Zhili Yang

**Affiliations:** ^1^ Department of Thyroid, Parathyroid, Breast and Hernia Surgery, Shanghai Jiao Tong University Affiliated Sixth People’s Hospital, Shanghai, China; ^2^ Department of General Surgery, Shanghai Jiao Tong University Affiliated Sixth People’s Hospital, Shanghai, China

**Keywords:** sc-RNA seq, papillary thyroid carcinoma, ecosystem, progression, heterogeneity

## Abstract

**Background:**

Despite extensive research, the papillary thyroid carcinoma (PTC) ecosystem is poorly characterized and, in particular, locoregional progression. Available evidence supports that single-cell transcriptome sequencing (Sc-RNA seq) can dissect tumor ecosystems.

**Methods:**

Tissue samples from one PTC patient, including matched primary tumor (Ca), lymph node (LN) metastasis, and paracancerous tissue (PCa), were subjected to Sc-RNA seq with 10×Genomics. Dual-label immunofluorescence and immunohistochemistry were used to confirm the existence of cell subtypes in a separate cohort.

**Results:**

11,805 cell transcriptomes were profiled, cell landscapes of PTC were composed of malignant follicular epithelial cells (MFECs), CD8^+^ and CD4^+^ T cells, B cells, vascular endothelial cells, fibroblasts and cancer-associated fibroblasts (CAFs). Between Ca and LN ecosystems, the proportions of MFEC and interstitial cells were similar, less than 1/25(229/6,694, 361/3,895), while the proportion of normal follicular epithelial cells (NFECs) and interstitial cells was > 2 in PCa (455/171). NFECs in PCa formed a separate cluster, while MFECs in Ca and LN exhibited a profound transcriptional overlap, and the interstitial cells among these samples had an overall concordance in their identity and representation, albeit with some distinctions in terms of the cell percentage per subset. A fraction of the B cell subpopulation in Ca expressed inhibitory receptors, while their respective ligand genes were clearly transcribed in T cell and malignant epithelial cell clusters, while some CD8^+^ T cells in both Ca and LN produced high levels of inhibitory receptors whose respective ligands were overexpressed in some CD4^+^ T cells. Three CAF subtypes in Ca and LN were identified, which may be due to mutual transitions.

**Conclusions:**

Our data provide new insights into the PTC ecosystem and highlight the differences in ecosystems in PTC progression, which updates our understanding of PTC biology and will improve individualized patient treatment.

## Introduction

Thyroid cancer is the most common malignant endocrine tumor, with rapidly increasing incidence globally over recent decades (1). As the most common histological type, papillary thyroid carcinoma (PTC) accounts for most new cases, and death mainly occurs in advanced-stage patients with regional and distant metastases (2). Therefore, an in-depth understanding of the biological characteristics of PTC progression is key to establishing effective treatments, reducing mortality, and improving prognosis.

Biological alterations during tumor progression arise in several crucial transitions, including tumor initiation, local expansion, metastasis, and therapeutic resistance, which involve complex interactions between cells within the dynamic tumor ecosystem (3). In PTC, the current understanding of ecosystem heterogeneity is primarily based on genomic and transcriptomic methods that have profiled them in bulk (4), providing critical information yet masking the diversity of cells within each tumor.

Single-cell transcriptome sequencing (Sc-RNA seq) is based on comprehensive and quantitative interpretation of mRNA information to identify individual cell identities, resolve tumor tissue heterogeneity, uncover gene regulatory relationships, and trace the transcriptional trajectories underlying malignant transformation (5). Sc-RNA seq has been used to dissect the cell landscapes in ecosystems such as liver cancer (6), head and neck cancer (7), breast cancer (8), melanoma (9), and pancreatic cancer (10). Recently, Sc-RNA seq analyses in mouse metastatic thyroid carcinoma have revealed the cellular landscape and focused on two subsets of follicular epithelial cells that perform regenerative functions (11), and in the zebrafish thyroid gland, thyrocyte diversity has been documented (12). We found that the *ARHGAP36* gene is exclusively expressed in thyroid malignant follicular epithelial cells (MFECs) by Sc-RNA seq analysis and further identified its function in promoting tissue invasion and tumor metastasis (13).

Here, we prepared viable single cells from a PTC patient diagnostically confirmed by pathology, including matched primary tumor (Ca), lymph node (LN) metastasis, and adjacent normal tissues (PCa). A total of 11,805 cells were subjected to Sc-RNA seq and cell landscapes were identified. Then, ecosystem differences involving the three matched samples were compared using aggregate analysis. Our data provide new insights into the ecosystem of PTCs and highlight differences in ecosystems in PTC progression, which updates our understanding of PTC biology and will improve individualized patient treatment.

## Materials and Methods

### Clinical Specimens

**Figure 1:**
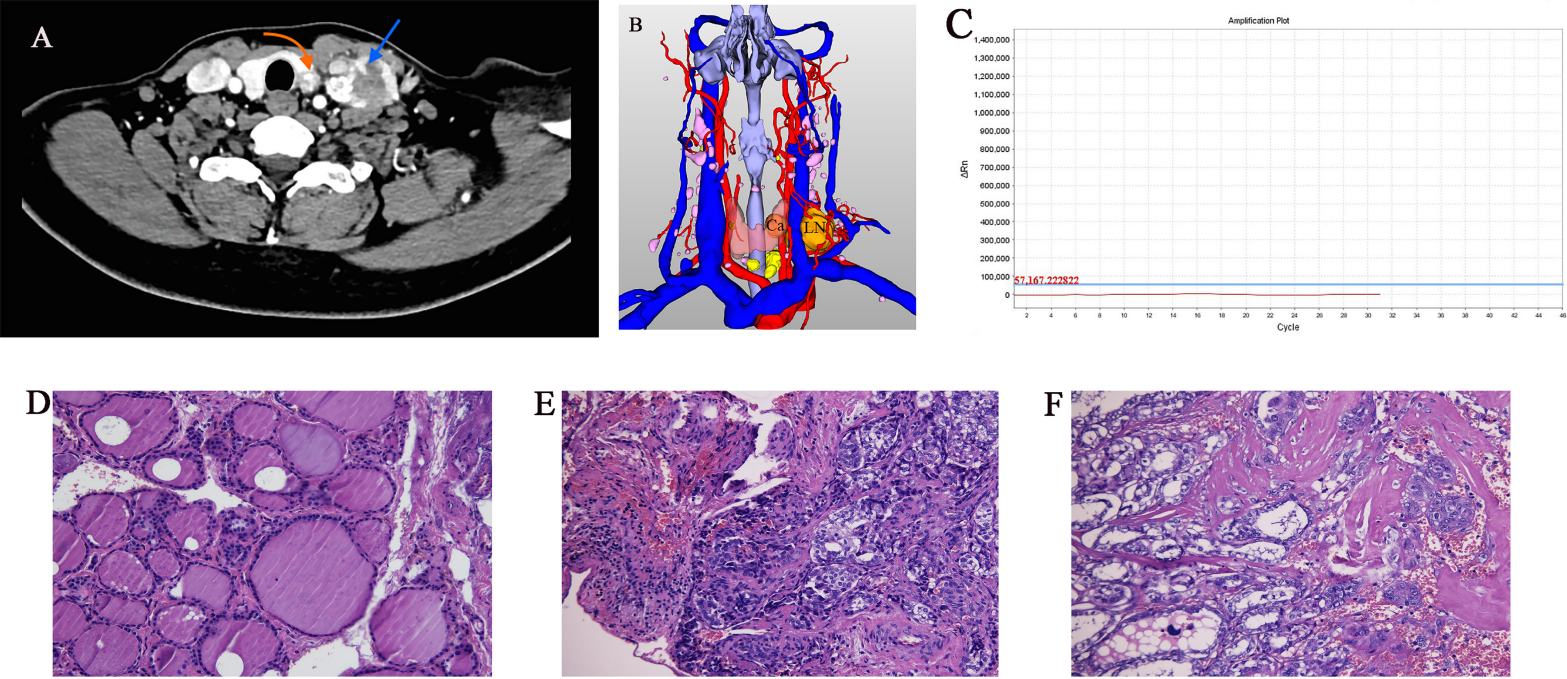
Imaging and histology of matched PTC cases (Ca, primary PTC samples; LN, lymph node metastasis sample). **(A)** Axial CT contrast-enhanced scan of neck showing primary and lymph node metastasis tumors (arrows). **(B)** Three-dimensional CT imaging of neck. **(C)** *BRAF* gene mutation test result was negative. **(D–F)** Pathology sections of tumor tissue stained with hematoxylin and eosin showing typical paracancerous tissue, primary PTC, and lymph node metastasis (original magnification 20×).

Primary PTC, lymph node metastasis, and matched adjacent normal tissue samples were harvested and cut into two parts during a surgical operation involving a 45-year-old female patient (**Figure 1**). Half of each tissue sample was completely immersed in MACS tissue preservation solution (Miltenyi Biotec, #130-100-008) and delivered to the laboratory within 2 h at 4°C, while the other section was fixed in paraformaldehyde for histological analysis. In addition, primary and metastatic carcinoma tissues from 10 patients with PTC were collected and fixed in 10% neutral formalin for immunostaining (**Supplementary Table 1**). None of the patients underwent any preoperative treatment. The pathological diagnoses of the samples were independently confirmed by two pathologists.

### Tissue Processing and Enrichment of Single Cells

The tissues were washed twice with PBS. The biopsy specimens were cut into 1 mm^3^ pieces using sterile scalpel blades and placed in Petri dishes. Sample dissociation was performed according to the instructions of a human tumor dissociation kit (Miltenyi Biotec, #130-095-929), using a gentleMACS Octo automatic tissue processor (Miltenyi Biotec, #130-096-427). Large lumps of tissue were removed by a membrane with a pore size of approximately 100 µm. The cells were then centrifuged at 300 × *g* for 5 min. The cells were resuspended in red blood cell lysis buffer, cultured at room temperature for 15 min, and then centrifuged at 120 × *g* for 3 min at 4°C. The remaining cells were diluted with PBS containing 0.04% BSA (Sigma) to achieve a concentration of approximately 10^6^ cells per microliter. Cell viability was assessed by 0.4% Trypan blue (Invitrogen) exclusion staining.

### Single Cell Capture, cDNA Library Preparation, and Sequencing

Single cell suspensions at a concentration of 300-600 living cells per microliter determined by Count Star were loaded onto a Chromium single cell controller (10×Genomics) to generate single-cell gel beads in an emulsion according to the manufacturer’s protocol. Using a S1000TM Touch Thermal Cycler (BioRad), cDNA was generated and then amplified, and the quality was assessed using Agilent TapeStation 4200 system. According to the manufacturer’s instructions, Single-cell RNA-seq libraries were constructed using Single Cell 3’ Library Gel Bead Kit V2 (10×Genomics, 120237). The cells were sequenced to a depth of at least 10^5^ reads per cell and 150 bp (PE150) paired-end reads on an Illumina Novaseq6000 sequencer (CapitalBio, Beijing, China).

### Bioinformatics

We used Cell Ranger v.3.0.2 (10×Genomics) to process raw sequencing data. Files from the Illumina Novaseq6000 were demultiplexed and converted to FASTQ files. Transcript counts for each cell were quantified using barcoded Unique molecular identifiers (UMIs) and 10× cell barcode sequences. Gene-by-cell-expression matrices were loaded into the R package Seurat v.3.0 for quality control and downstream analyses. For aggregation analysis, the combined data was aggregated from the Cell Ranger count data, normalized to the same sequencing depth, and recalculated the gene expression matrix. Qualified cells were retained based on genes with > 200 cells detected and cells with >500 and<10 000 genes and a mitochondrial gene percentage of <30%. Single-cell trajectories were built using the Monocle package (version 2.8.0). Dimensionality reduction was performed by visualization using t-distributed stochastic neighbor embedding (t-SNE) overlays and marker gene heatmaps. Cell clusters were identified using the FindClusters function based on a K-means clustering algorithm implemented in Seurat.

### Thyroid Histology and Immunostaining

Paraffin-embedded 3-µm-thick sections were stained with hematoxylin and eosin (H&E) according to routine histological protocols or deparaffinized and rehydrated. For immunostaining, antigen retrieval was performed using a pressure cooker for 15–20 min in 0.01 M citrate buffer (pH 6.0) to remove aldehyde links formed during initial fixation of tissues. Sections were then blocked in PBS containing 10% bovine serum albumin for 1 hour at room temperature. After blocking, one part of the samples was incubated with rabbit anti-human α-SMA/ACTA2 (1:200, Abcam, ab32575) and sheep-anti-human FAP (1:200, Affinity, AF3715) primary antibodies overnight at 4°C. Secondary antibodies (Invitrogen) were incubated for 1 h at room temperature. DAPI (1 μg/ml,Invitrogen) was then used to counterstain nuclei, and the remaining samples were incubated with primary antibodies against CD20 (1:200, Abcam) at 4°C overnight. The sections were then stained with the appropriate HRP-labeled polymer-conjugated secondary antibodies for 60 min. Immune complexes were visualized by exposure to DAB substrate for 3-5 min. Nuclei were counterstained with H&ERrepresentativeimmunostaining images from each specimen were captured and analyzed using Image-Pro Plus v.6.0.

### Statistics

Quantitative data are presented as means ± SEM, or means ± SD. Statistical analyses were performed using a two-tailed paired Student’s *t*-test. Statistical significance was set at p value < 0.05. For scRNA-seq analysis, 1-tailed Welch’s *t* test with p value < 0.01 was used for cell type-specific signature gene selection.

## Results

### Single-Cell Expression Atlas of PTC Progression

**Figure 2:**
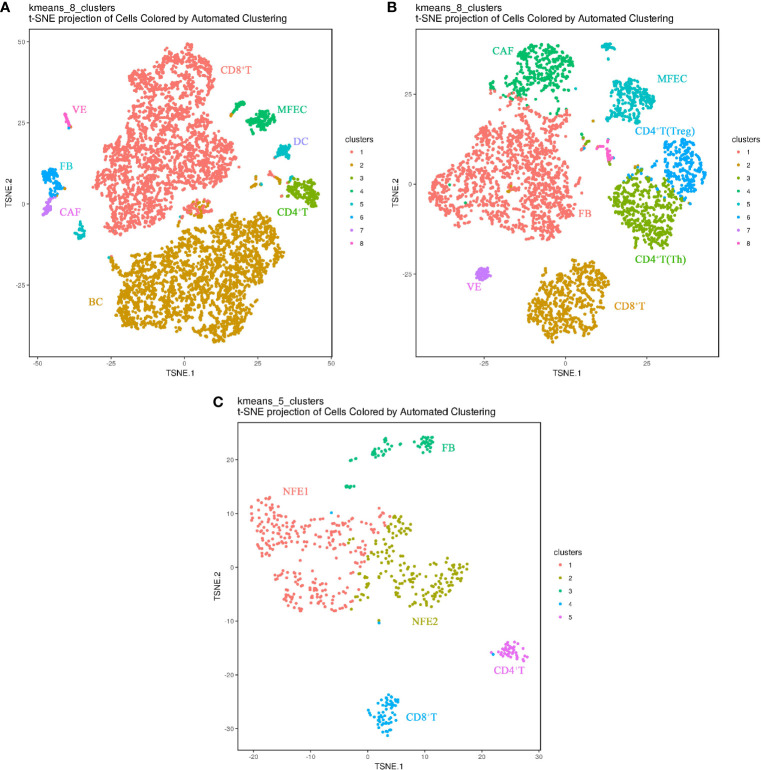
Cell landscapes of matched PTC samples by single-cell transcriptomic analysis(The closer together cells are plotted, the more similar they are; k-means cluster assignment is indicated by color; clusters are labeled based on expression of canonical marker genes). **(A)** t-SNE plot demonstrating eight main cell types in primary PTC(Ca). **(B)** t-SNE plot demonstrating eight main cell types in metastatic PTC(LN). **(C)** t-SNE plot demonstrating five main cell types in paracancerous normal tissue(PCa). NFE, Normal Follicular Epithelial cell; ME, Malignant Epithelial cell; VE, Vascular Endothelium cell; FB, Fibroblast cell; CAF, Cancer-associated fibroblast; DC, Dendritic cell; Treg, Regulatory T cell; Th, helper T cell; BC, B cell.

In the matched samples, 626 cells from adjacent normal tissues (PCa), 6923 cells from primary tumor (Ca), and 4256 cells from metastatic lymph node (LN) were analyzed by Sc-RNA seq, clustered, and then annotated with the identity of cell clusters(**Figures 2A–C**). Among them, normal follicular epithelial cells (NFECs) from PCa were identified by *TG*, *EpCAM*, *TPO*, etc. marker genes, malignant follicular epithelial cells (MFEC)s from Ca and LN by *TG*, *EpCAM*, *CK19/KRT19, MET*, etc. marker genes, and interstitial cell subsets from the three samples by their respective marker genes, such as CD4^+^ T (Th) cells (*CD4+, CD25^-^*)/CD4^+^ T (Treg) cells (*CD4^+^, CD25^+^*), CD8^+^ T cells (*CD8*), B cells (*CD19, CD20*), vascular endothelial cells (*VWF*, *ACKR1*), dendritic cells (*LAMP3*), fibroblasts (*ACTA2, FN1, THY1, TAGLN*), and CAFs (*FAP, POSTN, CXCL12*) (**Supplementary Figures S1A, B**). Normal follicular cell subsets were the main components of PCa (455/626), while MFEC subsets only accounted for a small proportion of Ca as well as LN (229/6,923, 361/4,256), which is the authentic cancer cell population in cancer tissues (**Figures 2A–C**).

### Intrinsic Differences of Ecosystems in Paracancerous, Primary, and Metastatic PTCs

**Figure 3:**
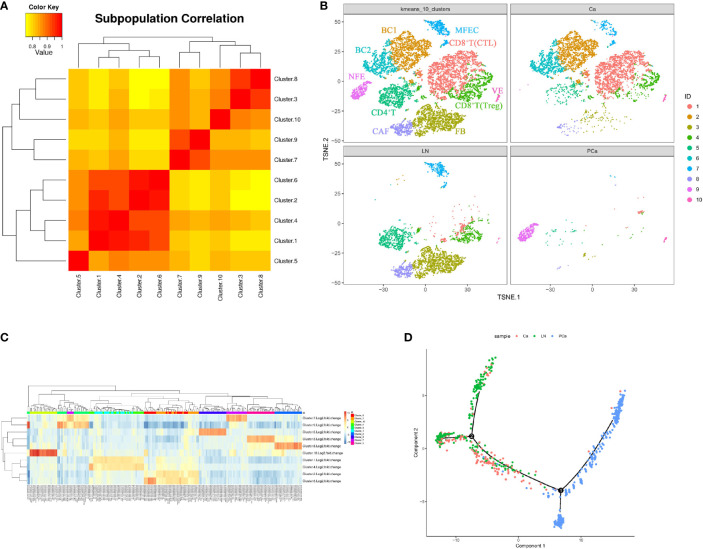
Cellular heterogeneity during PTC progression (Ca, primary PTC; LN, lymph node metastasis; PCa, paracancerous tissue). **(A)** Diagram of cell cluster correlation. **(B)** Aggregate analysis of ecosystems in paracancerous, primary and metastatic PTCs. The t-SNE plot of aggregate analysis from paracancerous, primary and metastatic PTCs, showing the formation of 10 main clusters shown in different colors. The functional description of each cluster is determined by the gene expression characteristics of each cluster. Cluster1: CD8+T(CTL); Cluster2: BC1; Cluster3: FB; Cluster4: CD8+T(Treg); Cluster5: CD4+T;Cluster6: BC2; Cluster7: MFEC; Cluster8: CAF; Cluster9: NFE; Cluster10: VE. **(C)** Heatmap illustrating expression levels of specific markers in each cell cluster. Detailed different genes were listed in **Supplementary Table 2**. Expression data are calculated as Log2 Fold Change. **(D)** Pseudotime PTC trajectory analysis. Each point corresponds to a single cell, and each color represents a sample. NFE, Normal Follicular Epithelial cell; ME, Malignant Epithelial cell; VE, Vascular Endothelium cell; FB, Fibroblast cell; CAF, Cancer-associated fibroblast; Treg, Regulatory T cell; Th, helper T cell; BC, B cell.

Aggregate analysis of all cells from the matched samples was further used to ascertain the relationships involving these cell populations and the dynamics of their phenotypic changes during PTC progression (**Figures 3A, B**). This showed that the NFECs in PCa formed a separate cluster, while MFECs in Ca and LN exhibited profound transcriptional overlaps. Interstitial cells among the matched samples exhibited an overall concordance in their identity and representation, albeit with some distinctions in terms of cell percentages per subset (**Figures 3B, C**). For example, BC1/mature B cells (CD19+, CD20+, CD23+/-) and BC2/activated B cells (CD19+, CD20+, CD23+) subsets were only found in Ca, and the cytotoxic T lymphocyte (CTL) subtypes (CD8+, CD28+) in CD8^+^ T cells were more concentrated in Ca. In contrast, there were more CD4^+^ T cells in LN, whereas fibroblasts and CAFs were significantly enriched in LN. In addition, there were few interstitial cells in PCa. The trajectory of differentiation commenced with follicular epithelial cells of PCa and ended with malignant epithelial cells of primary and metastatic PTCs, while their trajectory branches partly overlapped between primary and metastatic malignant cells (**Figures 3D**).

### Putative Interactions Between Immune Cell Types in PTC

**Figure 4:**
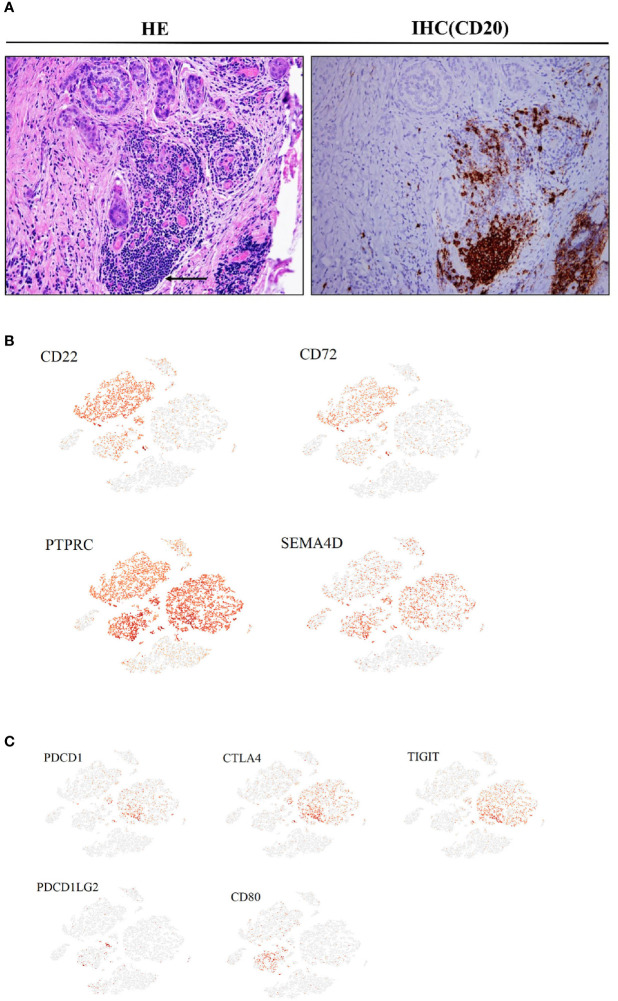
Cancer-associated immune cells and inhibitory regulation in PTC (Ca, primary PTC; LN, lymph node metastasis; PCa, paracancerous tissue). **(A)** IHC with CD20 antibodies in another PTC cohort(original magnification 20×). **(B)** Expression levels of CD22, CD72, PTPRC and SEMA4D on the t-SNE plot, with each cell colored based on the relative normalized expression. **(C)** Expression levels of PDCD1, CTLA4, TIGIT, PDCD1LG2 and CD80 on the t-SNE plot with each cell colored based on the relative normalized expression.

In primary tumor tissues, large numbers of mature and activated B cells emerge, expressing markers such as CD20 and CD19, although no plasma cells were observed. This was confirmed using immunohistochemistry (IHC) with anti-CD20 antibodies in another PTC cohort (**Figure 4A**). Some cells in this subset also expressed the inhibitory receptors CD22 and CD72, and their interacting ligands, PTPRC and SEMA4D, were clearly expressed in T cells and malignant epithelial cell clusters (**Figure 4B**). A fraction of CTLs in the CD8+ T cell subtype expressed high levels of PD1, CTLA4, and TIGIT inhibitory receptors in both primary and metastatic tumor samples, and their interacting ligand transcripts such as *PDCDILG2* and *CD80* were overexpressed in a small fraction of CD4+T cells, CAFs, and malignant cell subpopulations (**Figure 4C**).

### Diversity of CAFs in PTC

**Figure 5:**
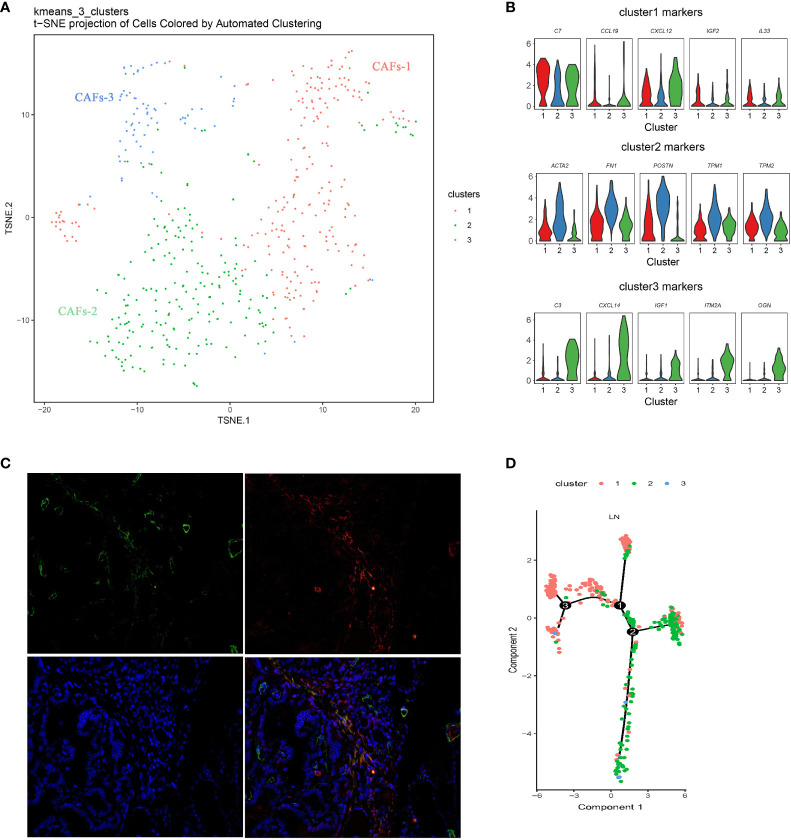
Diversity of CAFs in PTC **(A)** Reclustering of CAFs represented as a t-SNE plot. (The closer together cells are plotted, the more similar they are; k-means cluster assignment is indicated by color). **(B)** Violin plots of selected genes, showing normalized expression in the different subclusters. **(C)** Double immunofluorescent staining for ACTA2 and FAP in human metastatic PTC tumors. **(D)** Pseudotime analysis of CAFs in metastatic PTC. Each point corresponds to a single cell, and each color represents a cell cluster. LN, lymph node metastasis; CAF, Cancer-associated fibroblast.

There was a certain number of CAFs in primary and metastatic tissues, and three distinct molecular subtypes of CAFs were identified through finer-scale clustering (**Figure 5A**). CAFs-1 markedly expressed an abundance of chemokines, such as C77, CCL19, CXCL12, IL33, and IGF2. CAFs-2 specifically expressed FN1, TNFAIP6, POSTN, TPM1, TPM2, and the myofibroblast marker ACTA2. The two subsets mainly originate from LN. CAFs-3 from the primary tumor showed distinct expression of other chemokines and growth factors, such as C3, CCL5, IGF1, CXCL10, CXCL9, and CXCL14, in addition to CCL19 and IGF2 (**Figure 5B** and **Supplementary Figure S2**). Double immunofluorescent staining for ACTA2 and FAP demonstrated that CAFs-2 was present in human metastatic PTC tumors (**Figure 5C**). Pseudotime analysis showed that CAFs-1 cells and CAFs-2 cells were located under different transcriptional states on the one trajectory path in metastatic PTC (**Figure 5D**).

## Discussion

Despite extensive efforts to dissect the cellular composition of PTC (5), to the best of our knowledge, an accurate, intuitive cell landscape in PTC has not been achieved. After performing Sc-RNA seq of the present matched PTC samples, it was shown that a multicellular ecosystem of PTC was composed of MFECs and interstitial cells, and their detailed cell types were consistent with previously described types in bulk samples (5). However, it is notable that in primary and metastatic PTC ecosystems, the number of MFECs only accounted for a small proportion of total cells in Ca and LN, while there was a large proportion of follicular epithelial cells in normal tissues adjacent to cancerous tissues, which is consistent with the results of a recent Sc-RNA seq study involving adult thyroid tissue (14). The recruitment of large numbers of interstitial cells by cancer cells constitutes a unique ecosystem that may explain the indolent behavior of most PTCs.

To understand the intrinsic differences involving ecosystems in PTC progression, we performed aggregate analysis of all cells from matched samples. The results showed that the transcriptional profiles of MFECs in primary or metastatic PTC and NFECs in PCa tissue were significantly different. However, the transcriptional profiles of MFECs unexpectedly overlapped between primary and metastatic PTCs, which was consistent with earlier findings in primary and metastatic head and neck cancer (7). Combined with pseudotime analysis of differentiation trajectories of normal follicular cells and primary and metastatic cancer cells in the present case, this phenomenon changes our previous understanding of differential gene expression in metastatic and primary cancer cells in bulk, which returns to the common sense concept that metastatic cancer cells are derived from primary cancer cells, and supports the phenotypic plasticity of cancer cells from epithelial-mesenchymal transition (EMT) to mesenchymal-epithelial transition (MET)in the metastasis cascade (15, 16). In contrast to tumor parenchymal cells, the present single-cell analysis confirmed that there were differences in the ecology of primary and metastatic PTC cells. For example, B and CTL cells were concentrated in the microenvironment of primary cancer cells, whereas CD4^+^ T cells and CAFs were found in the microenvironment of metastatic cancer cells. Our findings suggest that the transition of cancer cells from a primary state to one progression may be provoked by changes in their local environment, and metastatic colonization requires, or at least can be aided by, a supportive immunity and extracellular matrix (15).

Our data showed that the number of regulatory T cells (T_regs_) was more widely distributed in the metastatic tumor microenvironment than in primary tissues. It has been recognized that cancer cells can evade destruction by cancer-attacking immune cells such as CTLs, aided by immunosuppressive T_regs_ that depend on a lipid production pathway in the tumor microenvironment (17). Therefore, it is suggested that T_regs_ play a key role in the growth of metastatic PTC cells, and targeting them may be a new alternative treatment strategy for metastatic PTC (18).

From the present data, a novel finding was that a large number of mature and activated B cell subsets exist in the primary PTC ecology, but not in normal and metastatic tissues. Mature and activated B cells are recognized as the main effector cells of humoral immunity, which suppress tumor progression by secreting immunoglobulins, promoting T cell responses, and improving survival (19). A recent study confirmed that tumor-infiltrating B cells cluster, HPV-specific antibody secreting cells (ASCs), can produce virus specific antigen-antibody responses to human head and neck cancer-specific papillomavirus (20). At the same time, our data showed that some of these B cells expressed inhibitory receptor genes, and their corresponding ligand molecules were displayed by T cells and malignant epithelial cells. Together, this evidence suggested that although the underlying mechanism of the difference between B cells aggregation in primary and metastatic foci is unclear, as an important member, B cells interact with other members of the PTC ecosystem to affect the state of tumor cells. The profound transcriptional overlap between primary and metastatic lesions our data showed may be the result of Spatial and temporal microenvironment homeostasis in PTC.

Our data revealed that immune checkpoint genes *PDCD-1, CTLA4*, and *TIGIT* were expressed in a small proportion of CTL cells in primary and metastatic PTCs, and most of their interacting ligands are expressed in the surrounding CD4+ T cells, suggesting that checkpoint blockade approaches that target these markers might be effective for PTC patient therapy. In fact, combinations of immune checkpoint inhibitors such as pembrolizumab and multikinase inhibitors such as lenvatinib as effective treatments have entered phase II clinical trials (21).

CAFs are considered one of the most abundant stromal cells in the ecosystems of almost all solid tumors, including PTCs (22). With the application of scRNA-seq technology, CAFs have been shown to have heterogeneity and plasticity and play several roles in the development of tumors, including promoting cancer cell proliferation, resistance to therapy, and immune exclusion, and restraining tumor progression (23). Current data show that myofibroblastic CAF (myCAF) subsets are concentrated in metastatic PTC. Since such CAFs are associated with an extracellular matrix signature, which is considered to contribute to therapy resistance (23), it is difficult to cure metastatic PTC foci, and targeting such cells may be an effective treatment. In addition, trajectory analysis showed that inflammatory CAFs and myCAFs are located on different transcriptional states on one trajectory path in metastatic PTC and inflammatory CAFs only in primary PTC, suggesting that there is a state transition between the two, which may be temporal and spatial gene expression differences for the microenvironment between primary and metastatic lesions.

Metastasis cascade of cancer cells includes the key stage of clonal growth at distant sites, and the characteristic of this stage is that cancer metastases tend to recapitulate significant epithelial features of their corresponding primary tumors (15). As in the present single-cell data, the transcription of metastatic cancer cells is consistent with that of their primary cancer cells. Two recent studies on Sc-RNA seq sequencing and lineage tracing show that cancer cells pre-metastasis have partial EMT (7) or hybrid EMT state (24), suggesting that metastatic cancer cells have their own reversible EMT genetic program. At the same time, in this single-cell data, the ecosystem of metastatic cancer, which is different from that of primary cancer cells, contributes to immunosuppression and stromal environment of pro-metastasis, indicating that it assists the transcriptomic expression of metastatic cancer cells.

In conclusion, we comprehensively dissected ecosystem differences during PTC progression. Our work suggests that metastatic PTC might benefit from targeted therapy for specific cell subtypes. Importantly, we identified the ecological characteristics of PTC that are different from those of previous bulk tissue studies, and anticipate that more datasets for PTC ecosystems will become available with emerging single-cell technologies.

## Data Availability Statement

The datasets presented in this study can be found in online repositories. The name of the repository and accession number can be found below: National Center for Biotechnology Information (NCBI) BioProject, https://www.ncbi.nlm.nih.gov/bioproject, PRJNA766250.

## Ethics Statement

The studies involving human participants were reviewed and approved by Ethics Committee of the Shanghai Jiao Tong University Affiliated Sixth People’s Hospital. The patients/participants provided their written informed consent to participate in this study. Written informed consent was obtained from the individual(s) for the publication of any potentially identifiable images or data included in this article.

## Author Contributions

ZY, GZ, and TY designed the study and performed the experiments. WQ and HW enrolled patients and collected samples. TY, WQ, and YF analyzed the data. TY, WQ, and ZY wrote and modified the manuscript. All authors contributed to the manuscript and have approved of the submitted version.

## Funding

This work was sponsored by Natural Science Foundation of Shanghai (21ZR1448500) and Hospital-level Clinical Research Fund of Shanghai Sixth People’s Hospital (YNLC201905).

## Conflict of Interest

The authors declare that the research was conducted in the absence of any commercial or financial relationships that could be construed as a potential conflict of interest.

## Publisher’s Note

All claims expressed in this article are solely those of the authors and do not necessarily represent those of their affiliated organizations, or those of the publisher, the editors and the reviewers. Any product that may be evaluated in this article, or claim that may be made by its manufacturer, is not guaranteed or endorsed by the publisher.

## Supplementary Material

Supplementary Figure S1 Expression levels of top20 genes in MEFCs **(A)** and NFECs **(B)** on the t-SNE plot, with each cell colored based on the relative normalized expression.

Supplementary Figure S2 Heatmap illustrating expression levels of specific markers in CAFs1-3.

## References

[1] Bray F Ferlay J Soerjomataram I Siegel RL Torre LA Jemal A. Global Cancer Statistics 2018: GLOBOCAN Estimates of Incidence and Mortality Worldwide for 36 Cancers in 185 Countries. CA: Cancer J Clin (2018) 68(6):394–424. doi: 10.3322/caac.21492 30207593

[2] Lim H Devesa SS Sosa JA Check D Kitahara CM. Trends in Thyroid Cancer Incidence and Mortality in the United States, 1974-2013. JAMA (2017) 317(13):1338–48. doi: 10.1001/jama.2017.2719 PMC8216772 28362912

[3] Rozenblatt-Rosen O Regev A Oberdoerffer P Nawy T Hupalowska A Rood JE. The Human Tumor Atlas Network: Charting Tumor Transitions Across Space and Time at Single-Cell Resolution. Cell (2020) 181(2):236–49. doi: 10.1016/j.cell.2020.03.053 PMC7376497 32302568

[4] Chmielik E Rusinek D Oczko-Wojciechowska M Jarzab M Krajewska J Czarniecka A. Heterogeneity of Thyroid Cancer. Pathobiology (2018) 85(1-2):117–29. doi: 10.1159/000486422 29408820

[5] Baslan T Hicks J. Unravelling Biology and Shifting Paradigms in Cancer With Single-Cell Sequencing. Nat Rev Cancer (2017) 17(9):557–69. doi: 10.1038/nrc.2017.58 28835719

[6] Sun Y Wu L Zhong Y Zhou K Hou Y Wang Z. Single-Cell Landscape of the Ecosystem in Early-Relapse Hepatocellular Carcinoma. Cell (2021) 184(2):404–21.e16. doi: 10.1016/j.cell.2020.11.041 33357445

[7] Puram SV Tirosh I Parikh AS Patel AP Yizhak K Gillespie S. Single-Cell Transcriptomic Analysis of Primary and Metastatic Tumor Ecosystems in Head and Neck Cancer. Cell (2017) 171(7):1611–24.e24. doi: 10.1016/j.cell.2017.10.044 29198524 PMC5878932

[8] Wagner J Rapsomaniki MA Chevrier S Anzeneder T Langwieder C Dykgers A. A Single-Cell Atlas of the Tumor and Immune Ecosystem of Human Breast Cancer. Cell (2019) 177(5):1330–45.e18. doi: 10.1016/j.cell.2019.03.005 30982598 PMC6526772

[9] Tirosh I Izar B Prakadan SM Wadsworth MH 2nd Treacy D Trombetta JJ. Dissecting the Multicellular Ecosystem of Metastatic Melanoma by Single-Cell RNA-Seq. Science (New York NY) (2016) 352(6282):189–96. doi: 10.1126/science.aad0501 PMC4944528 27124452

[10] Bernard V Semaan A Huang J San Lucas FA Mulu FC Stephens BM. Single-Cell Transcriptomics of Pancreatic Cancer Precursors Demonstrates Epithelial and Microenvironmental Heterogeneity as an Early Event in Neoplastic Progression. Clin Cancer Res (2019) 25(7):2194–205. doi: 10.1158/1078-0432.Ccr-18-1955 PMC6445737 30385653

[11] Hu A Yang LY Liang J Lu D Cao FF. Single-Cell RNA Sequencing Reveals the Regenerative Potential of Thyroid Follicular Epithelial Cells in Metastatic Thyroid Carcinoma. Biochem Biophys Res Commun (2020) 531(4):552–8. doi: 10.1016/j.bbrc.2020.06.050 32811644

[12] Gillotay P Shankar M Haerlingen B Sema Elif E Pozo-Morales M Garteizgogeascoa I. Single-Cell Transcriptome Analysis Reveals Thyrocyte Diversity in the Zebrafish Thyroid Gland. EMBO Rep (2020) 21(12):e50612. doi: 10.15252/embr.202050612 33140917 PMC7726803

[13] Yan T Qiu W Song J Fan Y Yang Z. ARHGAP36 Regulates Proliferation and Migration in Papillary Thyroid Carcinoma Cells. J Mol Endocrinol (2021) 66(1):1–10. doi: 10.1530/jme-20-0230 33112823

[14] Han X Zhou Z Fei L Sun H Wang R Chen Y. Construction of a Human Cell Landscape at Single-Cell Level. Nature (2020) 581(7808):303–9. doi: 10.1038/s41586-020-2157-4 32214235

[15] Lambert AW Pattabiraman DR Weinberg RA. Emerging Biological Principles of Metastasis. Cell (2017) 168(4):670–91. doi: 10.1016/j.cell.2016.11.037 PMC5308465 28187288

[16] Ganesh K Massagué J. Targeting Metastatic Cancer. Nat Med (2021) 27(1):34–44. doi: 10.1038/s41591-020-01195-4 33442008 PMC7895475

[17] Lim SA Wei J Nguyen TM Shi H Su W Palacios G. Lipid Signalling Enforces Functional Specialization of T(reg) Cells in Tumours. Nature (2021) 591(7849):306–11. doi: 10.1038/s41586-021-03235-6 PMC8168716 33627871

[18] Dees S Ganesan R Singh S Grewal IS. Regulatory T Cell Targeting in Cancer: Emerging Strategies in Immunotherapy. Eur J Immunol (2021) 51(2):280–91. doi: 10.1002/eji.202048992 33302322

[19] Bruno TC. New Predictors for Immunotherapy Responses Sharpen Our View of the Tumour Microenvironment. Nature (2020) 577(7791):474–6. doi: 10.1038/d41586-019-03943-0 PMC7523515 31965091

[20] Wieland A Patel MR Cardenas MA Eberhardt CS Hudson WH Obeng RC. Defining HPV-Specific B Cell Responses in Patients With Head and Neck Cancer. Nature (2021) 597(7875):274–8. doi: 10.1038/s41586-020-2931-3 PMC9462833 33208941

[21] Dierks C Seufert J Aumann K Ruf J Klein C Kiefer S. Combination of Lenvatinib and Pembrolizumab Is an Effective Treatment Option for Anaplastic and Poorly Differentiated Thyroid Carcinoma. Thyroid (2021) 31(7):1076–85. doi: 10.1089/thy.2020.0322 PMC8290324 33509020

[22] Ham J Wang B Po JW Singh A Niles N Lee CS. Cancer-Associated Fibroblasts (CAFs) in Thyroid Papillary Carcinoma: Molecular Networks and Interactions. J Clin Pathol (2021). doi: 10.1136/jclinpath-2020-207357 33619218

[23] Biffi G Tuveson DA. Diversity and Biology of Cancer-Associated Fibroblasts. Physiol Rev (2021) 101(1):147–76. doi: 10.1152/physrev.00048.2019 PMC7864232 32466724

[24] Simeonov KP Byrns CN Clark ML Norgard RJ Martin B Stanger BZ. Single-Cell Lineage Tracing of Metastatic Cancer Reveals Selection of Hybrid EMT States. Cancer Cell (2021) 39(8):1150–62.e9. doi: 10.1016/j.ccell.2021.05.005 34115987 PMC8782207

