# Supplementary figures and images for: Single-Cell Transcriptomic Analysis of Ecosystems in Papillary Thyroid Carcinoma Progression
Source: Front Endocrinol (Lausanne). 2021 Nov 1;12:729565. doi: 10.3389/fendo.2021.729565 (PMC8591202; doi:10.3389/fendo.2021.729565)

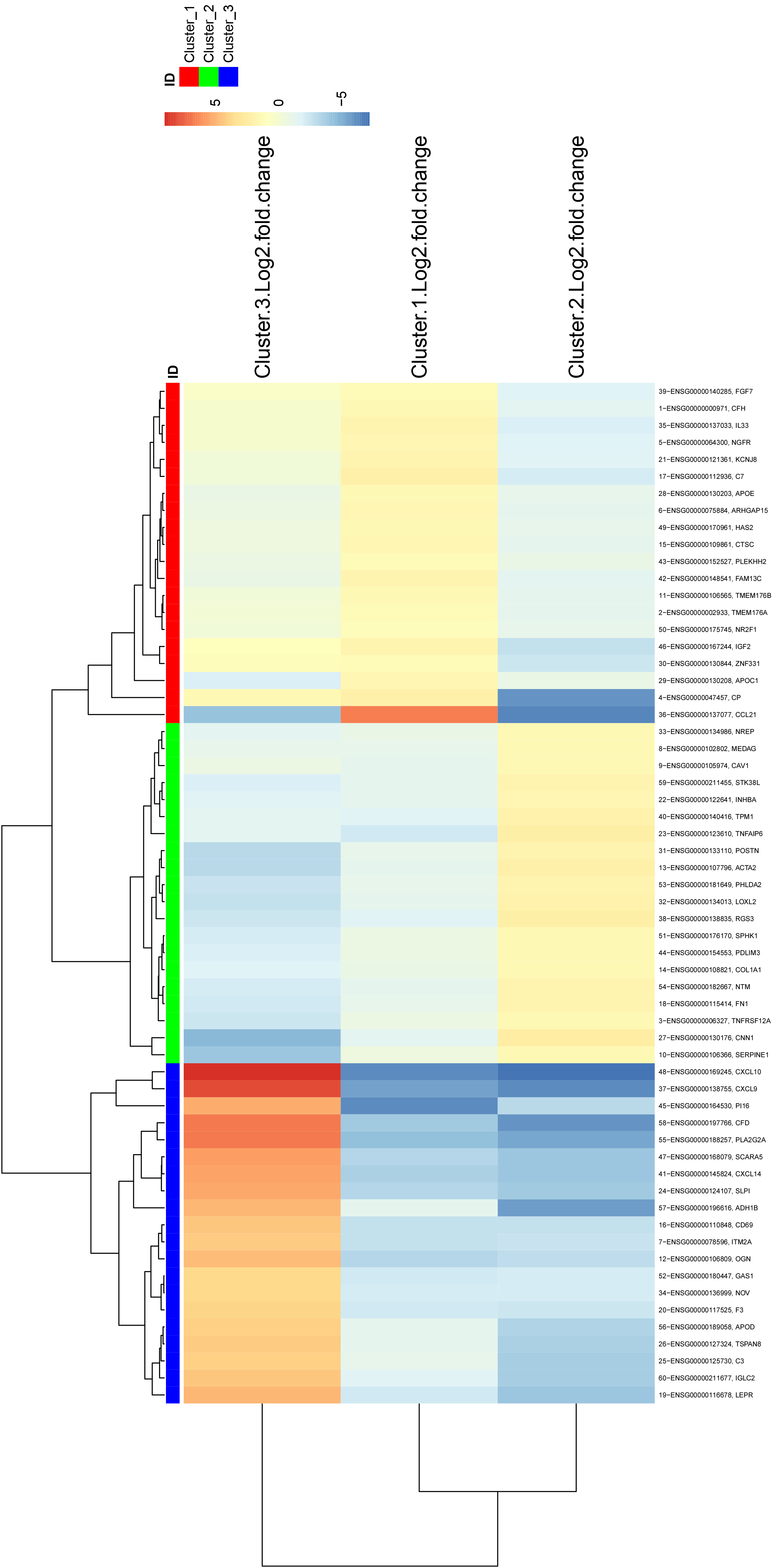

Supplement: Supplementary Figure S1 — Expression levels of top20 genes in MEFCs (A) and NFECs (B) on the t-SNE plot, with each cell colored based on the relative normalized expression. [file DataSheet_1.zip › Supplementary Figure/2.png]
